# Depletion of the Chromatin Looping Proteins CTCF and Cohesin Causes Chromatin Compaction: Insight into Chromatin Folding by Polymer Modelling

**DOI:** 10.1371/journal.pcbi.1003877

**Published:** 2014-10-09

**Authors:** Mariliis Tark-Dame, Hansjoerg Jerabek, Erik M. M. Manders, Dieter W. Heermann, Roel van Driel

**Affiliations:** 1 Swammerdam Institute for Life Sciences, University of Amsterdam, Amsterdam, The Netherlands; 2 Institute for Theoretical Physics, Heidelberg University, Heidelberg, Germany; 3 Van Leeuwenhoek Centre for Advanced Microscopy, University of Amsterdam, Amsterdam, The Netherlands; 4 Department for Molecular Biotechnology, University of Gent, Gent, Belgium; CNAG - Centre Nacional d'Anàlisi Genòmica and CRG - Centre de Regulació Genòmica, Spain

## Abstract

Folding of the chromosomal fibre in interphase nuclei is an important element in the regulation of gene expression. For instance, physical contacts between promoters and enhancers are a key element in cell-type–specific transcription. We know remarkably little about the principles that control chromosome folding. Here we explore the view that intrachromosomal interactions, forming a complex pattern of loops, are a key element in chromosome folding. CTCF and cohesin are two abundant looping proteins of interphase chromosomes of higher eukaryotes. To investigate the role of looping in large-scale (supra Mb) folding of human chromosomes, we knocked down the gene that codes for CTCF and the one coding for Rad21, an essential subunit of cohesin. We measured the effect on chromosome folding using systematic 3D fluorescent in situ hybridization (FISH). Results show that chromatin becomes more compact after reducing the concentration of these two looping proteins. The molecular basis for this counter-intuitive behaviour is explored by polymer modelling usingy the Dynamic Loop model (Bohn M, Heermann DW (2010) Diffusion-driven looping provides a consistent framework for chromatin organization. PLoS ONE 5: e12218.). We show that compaction can be explained by selectively decreasing the number of short-range loops, leaving long-range looping unchanged. In support of this model prediction it has recently been shown by others that CTCF and cohesin indeed are responsible primarily for short-range looping. Our results suggest that the local and the overall changes in of chromosome structure are controlled by a delicate balance between short-range and long-range loops, allowing easy switching between, for instance, open and more compact chromatin states.

## Author Summary

Folding of chromosomes in interphase nuclei of higher eukaryotes is a key element in regulating gene expression. The mechanisms that control chromatin folding are largely unknown. We have shown earlier that looping is a fundamental aspect of large-scale chromatin structure. Two abundant looping proteins are known: CTCF and cohesin. Here we combine quantitative fluorescent in situ hybridisation experiments in human cells with polymer modelling to unravel mechanisms of chromatin folding. We show that chromatin becomes more compact after depletion of looping proteins. This is remarkable, since polymer models describing chromatin predict decompaction. We present a polymer model that shows that specific biologically relevant looping regimes give rise to this behaviour. Importantly, chromosome conformation capture studies of mammalian chromatin support such looping regimes. Our results indicate that the local and overall compaction of the chromatin is defined by a subtle balance between short and long range loops; this may explain cell cycle and genome activity dependent structural transitions of chromatin.

## Introduction

Chromosomes are highly folded inside the interphase nucleus. Their structure has been extensively studied by light and electron microscopy and more recently by genome-wide mapping of intra- and inter-chromosomal contacts. The picture that is emerging is that the chromosomal fibre is packed in a hierarchical way on different length scales [1]–[3]. Complete chromosomes are confined to chromosome territories (CTs) that intermingle only to a limited extent [4]–[6]. Another well-defined level is that of the topologically associated domains (TADs), which are distinct sub-chromosomal structures in the Mb range [7]–[10]. These two levels of chromosomal organisation are evolutionary conserved in metazoans. Chromosomal folding is intimately linked with genome function. TADs are functional genomic units, each containing genes that often are transcriptionally or epigenetically co-regulated [2], [9]. Specific sequence elements between TADs, called insulators, confine long-range regulatory interactions between for instance enhancers and promoters to the individual TADs [11]. TADs coincide with DNA replication units, which show a distinct pattern of replication timing that is remarkably well conserved between mouse and man [12]–[15]. Another striking aspect of chromatin folding is the considerable cell-to-cell variation observed in populations of otherwise identical cells [16], [17]. Overall, chromosomal architecture seems a mix of well-defined and probabilistic components.

Despite the extensive information about chromosomal architecture and its importance for the functioning of the genome, underlying principles that direct chromosome folding are still elusive. What controls the dynamic folding of chromosomes? Such fundamental insight can be captured and explored in predictive computational models [18]–[21]. Models help to identify critical experiments and make sure that proposed mechanisms are physically and thermodynamically sound. Ideally, models should correctly reproduce the hierarchical architecture of interphase chromosomes, the probabilistic aspects of chromatin folding, as well as the structural transitions that chromosomes undergo during mitosis, meiosis and cell differentiation. Importantly, models should be able to make predictions that can be experimentally tested. Since chromosomal fibres are long flexible one-dimensional structures, polymer models are a good first approximation for chromosomes. Recently, a variety of such models have been proposed based on information from genome contact maps [20], [22]–[24]. So far, many models have not yet been thoroughly tested experimentally, which makes it difficult to assess their quality.

Recently, the Dynamic Loop polymer model (DL model) has been proposed, based on systematic 3D fluorescent *in situ* hybridisation (FISH) measurements on primary human fibroblasts, in combination with basic polymer physics [16], [25]. The DL model is based on first principles and its main parameter is the looping probability, describing the chance that two non-adjacent monomers of the polymer make contact, i.e. form a loop. The DL model shows that a linear polymer with randomly positioned loops with a broad length distribution correctly recapitulates three basic aspects of interphase chromatin: (i) the formation of chromosome territories, due to the entropy-driven segregation of looped chromosomes, (ii) the presence of discrete sub-chromosomal domains that differ in chromatin compaction due to local differences in looping frequency, and (iii) the considerable cell-to-cell variation in chromatin folding [25]–[28]. In the DL model diffusional behaviour of the chromatin fibre, which is considerable in interphase nuclei [29], drives the dynamic formation of loops. Importantly, the DL model is compatible with results of genome-wide intra-chromosomal contact mapping experiments [30]. The model indicates that the distribution of loops along the chromosome controls chromatin folding. To unveil further principles of chromosome folding we now test predictions of the DL model by manipulating the chromosome looping frequency and measuring the effect on chromosomal structure.

Two abundant nuclear proteins that are involved in chromatin looping are CTCF and cohesin [31]–[33]. Their binding sites on the genome in part co-localise indicating that they functionally cooperate [34], [35]. Reducing the levels of CTCF and Rad21, an essential component of the cohesin complex, in the cell has been shown to reduce the number of chromatin loops [36]–[38]. Here we analyse by quantitative 3D FISH the effect of CTCF and/or Rad21 knockdown on chromosome structure in human primary G1 fibroblasts. The DL model, similar to most other polymer models, predicts that upon reduction of the number of chromatin loops chromosomes become less compact. In striking contrast to this prediction our experiments show that they become considerably more compact after knocking down CTCF and Rad21. These observations put major constraints on polymer models that aim to recapitulate the behaviour of interphase chromosomes. We show that the DL model can be adapted to correctly describe the observed compaction. Systematic model simulations strongly suggest that the looping regime, i.e. loop frequency along the chromosomal fibre and loop size distribution, is the key variable that controls chromosome folding. The cell is able to manipulate chromosome folding by regulating the activity and concentration of looping proteins, such as CTCF and cohesin. The model makes specific predictions that are supported by recent experiments of others, underscoring the potential of the DL model to explore underlying principles of chromosome folding. Predictions of the adapted DL model about structural transitions that chromosomes undergo during cell differentiation, mitosis and meiosis are briefly discussed.

## Results

Basic polymer models describe the relationship between the mean square physical distance (MSD) of two monomers of the polymer (<R^2^>) and their distance along the polymer, the contour distance (also referred to as contour length in our previous work), expressed in the number of monomers (N). Experimentally, such a relationship can be measured for chromatin fibres in nuclei of fixed cells by FISH, using two probes that bind at a known genomic distance g (expressed in Mb) on the same chromosome. Confocal microscopy allows measuring their physical distance R (µm) in 3D. Systematic 3D FISH permits critical comparison between model predictions and experimental observations. This approach resulted in the Dynamic Loop (DL) model, which predicts that a limited number of quasi randomly distributed loops on a chromatin fibre is sufficient to confine a chromosome to a chromosome territory [16], [25]. The loop-size distribution observed in the DL model is in good agreement with what is observed in HiC experiments [30]. Moreover, the DL model shows that local differences in loop frequency along the chromatin fibre cause local differences in chromatin compaction, resulting in the formation of topological domains [27]. These and other approaches put chromatin looping on the central stage in controlling and switching large-scale chromatin structure in interphase nuclei. This implies that interfering with loop formation is likely to significantly affect chromatin structure. We tested this prediction by knocking down two proteins that play a major role in chromatin looping: CTCF and Rad21, an essential component of cohesin. The DL model predicts that a reduction of looping frequency results in chromatin expansion.

### Interfering with chromatin looping

**Figure 1:**
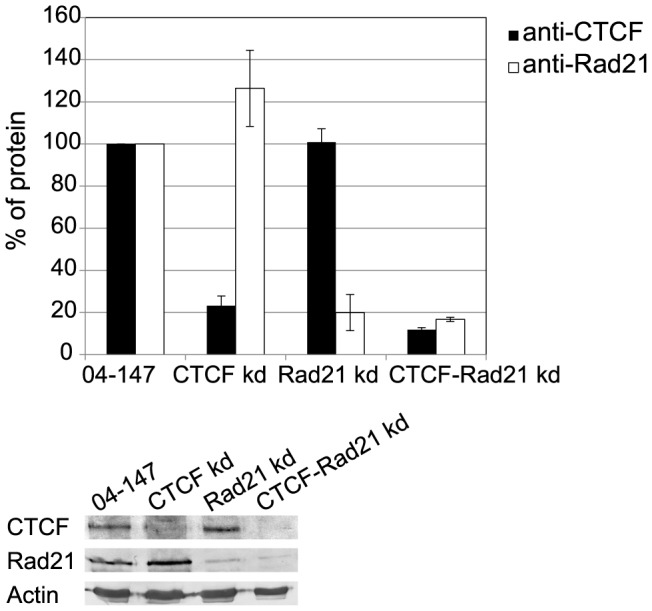
Knock-down of CTCF and Rad21. The nuclear fraction of control cells (04-147) and cells depleted of CTCF, Rad21 or both simultaneously were analysed by SDS-PAGE followed by immunoblotting. β-Actin was used as a control when calculating changes in CTCF and Rad21 protein levels after siRNA-mediated knock down. Average protein amounts of at least three independent experiments are shown. Error bars represent standard deviation.

The expression levels of CTCF and of cohesin were reduced by siRNA-mediated gene knockdown. To interfere with cohesin function we depleted its kleisin subunit Rad21. In the absence of this subunit none of the other cohesin subunits bind to chromatin [34]. The decrease in protein levels was quantified by Western blotting of nuclear fractions. Figure 1 shows that knockdown of CTCF and cohesin individually and in combination results in a decrease of ∼80% of their cellular concentration. A variety of studies have shown that such decrease in CTCF and cohesin significantly reduces chromatin looping [39], [40], but has no significant effect on cell cycle progression [41]. All experiments were carried out with human primary female fibroblasts to avoid possible effects of immortalisation. Depletion of CTCF and/or cohesin had no measurable effect on the cellular and nuclear morphology and the size of the nucleus during the time course of the experiments, i.e. up to 72 hours after siRNA transfection (Figures S1 and S2). Also the viability of cells was not affected and the percentage of apoptotic cells among cells analysed did not change, as demonstrated by AnnexinV staining (Figure S3)

### Depletion of CTCF and cohesin results in chromatin compaction

**Figure 2:**
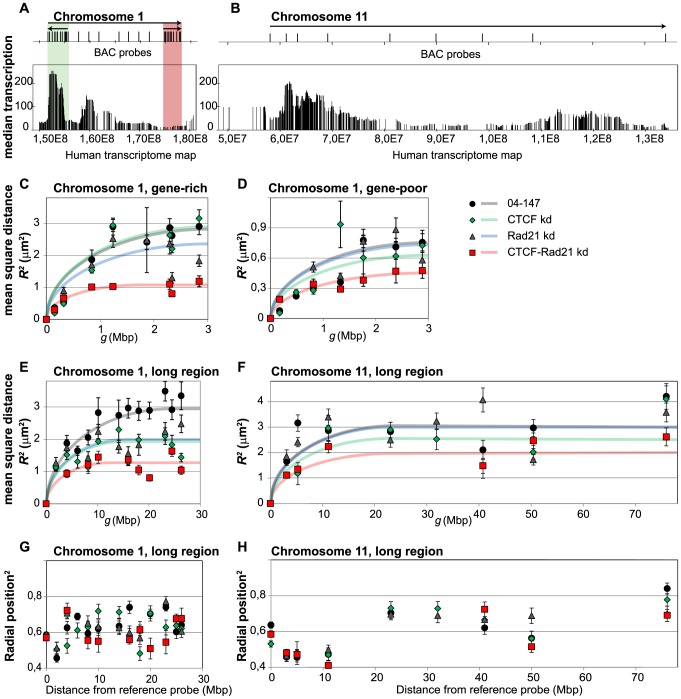
*In situ* chromatin structure. (**A, B**) The genomic context of analysed regions of the q-arms of human chromosome 1 (**A**) and chromosome 11 (**B**) is illustrated by the human transcriptome map. Vertical lines represent protein coding genes, the length depicting the average transcription level in a number of human tissues and cell lines [42]. The location of BAC probes used for FISH labelling are indicated above the maps. Arrows above the maps designate the regions where spatial distances between pairs of BAC probes were measured; the start of the arrow indicating the position of the reference BAC probe. On chromosome 1q three regions were analysed. Two 3 Mb regions were selected in a gene-rich and transcriptionally active region (green box) and in a region characterized by low gene density and low transcription (red box). (**C–F**) Graphs show the mean square physical distances <*R* ^2^> between the FISH probes as a function of the genomic distance *g* for a 3 Mb gene-rich (**C**) and a gene-poor (**D**) region on chromosome 1 q-arm. Longer regions of chromosome 1 (30 Mb) (**E**) and of chromosome 11 (76 Mb) (**F**) were also analysed. Each data point represents an average of 30–150 distance measurements in at least two independent experiments. Error bars represent standard errors. Graphs are shown for control cells (black dots) and cells after knockdown of CTCF (green diamonds), of cohesin subunit Rad21(blue triangles) and of CTCF and Rad21 simultaneously (red squares). Results show that chromatin becomes more compact after knockdown of CTCF and Rad21 (cohesin). (**G, H**) Radial positions of BAC probes in nuclei for chromosome 1q (G) and chromosome 11q (H). Radial nuclear position of a BAC probe was calculated the distance between the centre of gravity of a FISH labelled genomic site and the centre of gravity of the nucleus and dividing that distance with the length of a straight line drawn from the nuclear centre to the nuclear envelope through the centre of the gravity of the labelled site. Error bars represent standard errors. Note that some chromatin regions were poorly labelled in CTCF and/or cohesin knock down conditions and therefore omitted from the graph.

The relationship between the mean square physical distance (MSD) <R^2^> and the genomic distance g between FISH probes was determined for the same genomic regions as studied earlier in establishing the DL model, i.e. the q-arms of chromosomes 1 and 11 [16]. We concentrated on an about 3 Mb size gene-rich region gene-poor region of similar length on chromosome 1 and on a 27 Mb and a 70 Mb region on the q-arms of chromosome 1 and 11, respectively. The latter spans the complete q-arm of chromosome 11. Figures 2A and 2B show the analysed regions and the distribution of the BAC (bacterial artificial chromosome) probes used for FISH labelling. The BACs used in this study and the number of FISH probe-pairs analysed is listed in Table S1. Figure S6 shows the positions of the BACs on the genomic maps of the relevant parts of chromosomes 1 and 11 and relates them to the human transcriptome map [42], replication domains [43] and the HiC topological domains of similar cell types [7].

Only cells in G1 were used for these analyses. In the randomly growing cell culture S-phase cells were identified based on their incorporation of bromodeoxy uridine (BrdU) during DNA replication and G2 cells could be recognised by their significantly larger size [16], [44].

Figures 2C and D show the relationship between the MSD of two FISH probes and their genomic distance in two about 3 Mb regions on chromosome 1q, one being gene-rich (green) and the other gene-poor (red). Results from nuclei of control cells and of cells in which CTCF and cohesin were knocked down either individually or simultaneously are shown. Simultaneous knockdown of the two proteins resulted in considerable chromatin condensation, as shown by the decrease of the MSD plateau levels. The maximum average distances between the FISH probes decreased from about 2.7 µm to 1 µm for the gene-rich regions and from about 0.7 µm to close to 0.5 µm for the gene-poor regions. Knockdown of CTCF and cohesin individually had a considerably smaller effect. Control experiments using non-target siRNA did not reveal significant changes in chromatin compaction (Figure S4). Chromatin condensation is particularly evident for gene-rich areas, which are relatively open in control cells, and is smaller for the gene-poor regions that already are relatively compact in untreated cells. Figures 2E and F show the same type of measurements for the longer genomic stretches: 27 Mb of chromosome 1q and 70 Mb of chromosome 11q. At these longer distances the decrease of CTCF or of cohesin concentration reduced the maximum probe distance from about 3 µm to around 2 µm for chromosome 1q, indicating chromatin condensation, as found for shorter distances. Knockdown of both proteins simultaneously reduced the average distance further to close to 1 µm, indicating a three-fold compaction. For the 70 Mb region of chromosome 11q the effect of knocking down of CTCF and cohesin individually is less pronounced. The maximum average probe distance is about 3 µm, similar to the value for the chromosome 1q region in control cells. Simultaneous knockdown of CTCF and cohesin reduced this value to between 2 and 2.5 µm. What causes this quantitative difference in the behaviour of the two chromosomes is unclear. The average frequency of CTCF binding sites in both genomic regions is similar [45]. Importantly, depletion of the two looping proteins does not result in decondensation of chromatin, in contrast to what is predicted by most chromatin-inspired polymer models, including the DL model. Instead, after CTCF and cohesion depletion the opposite is observed, i.e. condensation of chromatin.

### The radial distribution of chromatin in the nucleus does not change after CTCF and cohesin knockdown

Although depletion of CTCF and cohesin causes the analysed regions to become more compact than in control cells, there are no evident changes in chromatin density when comparing DAPI staining signals of these cells (Figure S1). To find out if compaction of chromatin in CTCF and cohesin depleted cells results in large-scale chromatin rearrangements in in the nucleus that remain unseen in DAPI staining, we analysed the radial position of probes used for 3D FISH measurements in the nucleus. No significant changes in radial distribution of sites on the q-arms of chromosomes 1 and 11 were observed (Figures 2G and H), indicating that depletion of CTCF and cohesin has no effect on overall nuclear organization. The position of a locus in nuclear space and its compactness are correlated: gene-rich and open chromatin regions on average are located closer to the nuclear centre, whereas compact and gene-poor regions are located closer to the nuclear periphery [44], [46], [47]. Figure 2G shows that the analysed regions on chromosome 1q have approximately the same radial position. On chromosome 11 two well-defined regions can be identified (Figure 2H): the probes that label the centromere-proximal 0–11 Mb of the analysed region are closer to the nuclear centre, while the probes marking the 23–76 Mb area are located more towards nuclear periphery. This 23–76 Mb area of chromosome 11 is closer to the nuclear periphery compared to the probes on chromosome 1. In combination with measurements of relative volume of these regions carried out previously [44]. these results indicate that in control cells the 23–76 Mb domain of chromosome 11 has a more compact structure than the 0–11 Mb region on chromosome 11 and the analysed regions on chromosome 1.

### Polymer modelling of interphase chromatin

Recently, we proposed a simple polymer model that explains the confinement of an interphase chromosome to its chromosome territory. In this DL model the chromosomal fibre is represented as a self-avoiding random walk polymer forming probabilistic intra-polymer contacts between non-adjacent monomers [25]. As a consequence, loops with a broad size distribution are formed. The main model parameter is the looping probability (p), which is a measure for the probability that a bond (loop)is formed between two non-adjacent monomers is formed. Due to the proximity of monomers, short-range loops are formed more frequently than long-range ones, yielding a loop size distribution comparable to that observed in HiC experiments [30]. In the DL model a decrease in looping probability results in an increase of the volume occupied by the polymer, i.e. decondensation. Interestingly, our experimental results show that chromatin in interphase nuclei behaves in an opposite way, i.e. a decrease in chromatin looping due to depletion of CTCF and cohesin results in compaction. In the DL model decreasing the looping probability affect loops of all lengths. However, in cells CTCF seems involved preferentially in mediating short-range loops, i.e. below 1 Mb length [48].

**Figure 3:**
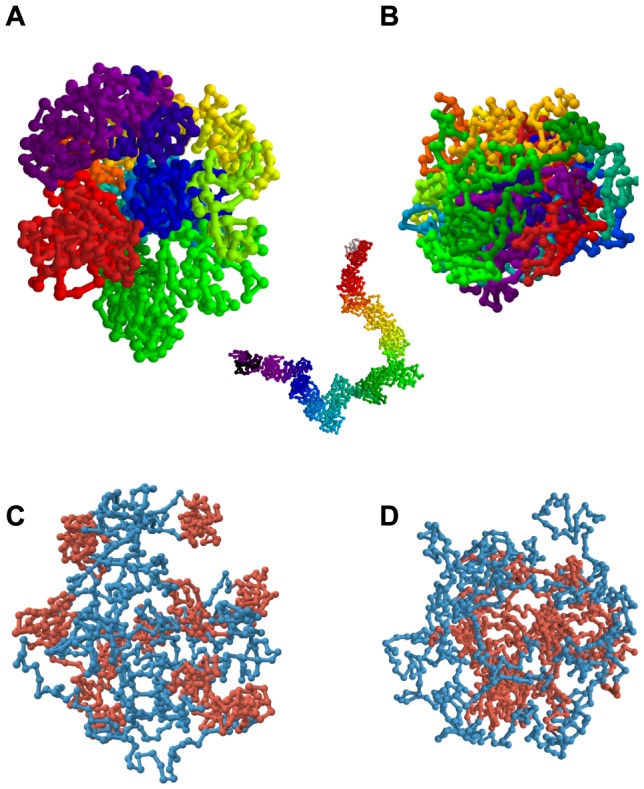
Example of polymer conformations of the adapted DL model at different looping regimes. (**A**) Conformation of the adapted-DL polymer with high short-range (p_short_ = 0.12) and low long-range (p_long_ = 0.04) looping probabilities and (**B**) the same polymer with low short-range (p_short_ = 0.04) and high long-range (p_long_ = 0.12) looping probabilities. The colour code labels the monomers of the polymer according to the visible spectrum along the length of the polymer. The inset in panel A displays the same situation as in (A) after abolishing all long-range looping (p_long_ = 0), showing that a uniform thick fibre is formed. More adapted DL polymer conformations with the same looping probabilities as shown in (A) and (B) are shown in Figure S5. (**C**) Conformation of the domain-adapted DL polymer with high short-range (p_short_ = 0.16) and low long-range (p_long_ = 0.02) looping probabilities and (**D**) the same polymer with low short-range (p_short_ = 0.02) and high long-range (p_long_ = 0.16) looping probabilities. Here, topological domains are labelled red and non-looping linker regions blue.

**Figure 4:**
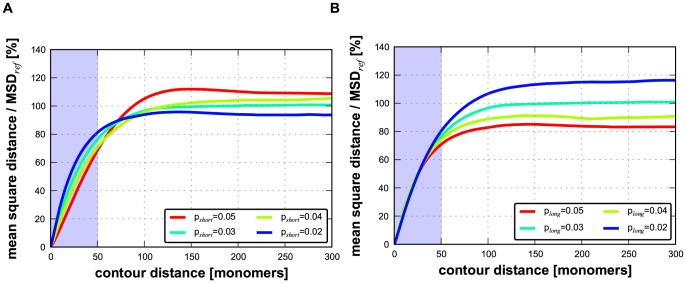
Effect of short-range and long-range looping probabilities on the relationships between the MSD and the contour distance in the adapted DL polymer model. (**A**) Varying the short-range looping probability at constant long-range looping probability (p_long_ 0.03). (**B**) Varying long-range looping probability at constant short-range looping (p_short_ 0.03). The MSD plateau levels is a measure of overall polymer compaction. The simulations show that decreasing the long-range looping probability results in expansion of the polymer, while lowering short-range looping probability leads to compaction. To facilitate comparison between the two effects, the MSD was normalized by the plateau level of the MSD for p_long_ = 0.03 and p_short_ = 0.03 (MSD_ref_) because this parameter combination is present in both figures.

**Figure 5:**
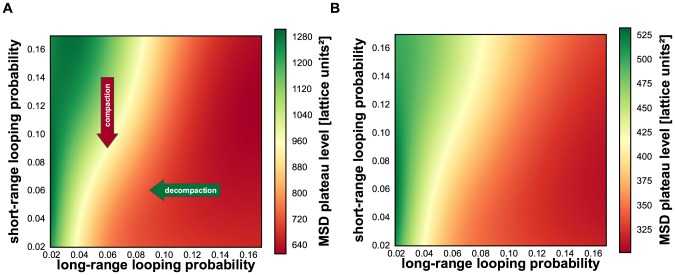
Heat plots showing how polymer compaction varies in the p_short_ - p_long_ parameter space. Polymer compaction is represented as MSD plateau level as shown in Figure 4. (**A**) adapted DL polymer, (**B**) domain-adapted DL polymer. The arrows in (A) show that decreasing the short-range looping probability (red arrow) results in compaction, while reducing the long-range looping probability (green arrow) leads to de-compaction. In the heat plot red marks compaction, green decompaction.

To explore whether the observed chromatin compaction may be caused by the predominant depletion of short-range loops, we modified the DL model by imposing different looping probabilities for short-range and long-range looping (p_short_ and p_long_, respectively). In simulations with this adapted DL model we used polymers with a total length of 1050 monomers (for further details see Materials and Methods section). Short-range loops were defined as those spanning 50 monomers or less and long-range loops as those spanning 51 monomers or more. For comparison, for human chromosomes in sizes 50–250 Mb the cut-off between short- and long-range loops would be approximately 2.5–12.5 Mb. Subsequently, we calculated the MSD *versus* contour distance relationship for a matrix of combinations of short- and long-range looping probabilities. Figure 3A illustrates the typical compact conformation of an adapted DL polymer with high p_short_ (0.12) and low p_long_ (0.04) probabilities. The colour code labels the monomers along the polymer according to the visible spectrum along the polymer. The inset in panel A displays the conformation for the same p_short_ (0.12) after p_long_ has been set to zero, showing that a high probability of the short-range interactions (p_short_) in the absence of long-range interactions results in a uniform thick fibre. Figure 3B shows a configuration of the DL polymer with low p_short_ (0.04) and high p_long_ (0.12) probabilities, showing a more chaotic folding of the polymer compared to Figure 3A. Figures 4A and B exhibit examples of the calculated relationships between the MSD and the contour distance, covering a range of p_short_ values (0.02–0.05), while keeping p_long_ constant (0.03) (Figure 4A), and of p_long_ (0.02–0.05) values at constant p_short_ (0.03) (Figure 4B). The plateau level MSD value is used as a measure for the overall compaction of the polymer: a lower MSD values indicates a stronger compaction. Remarkably, these simulations show that decreasing the long-range looping frequency p_long_ results in expansion of the polymer, while decreasing p_short_ leads to compaction. The heat map of Figure 5A displays the MSD plateau level of the polymer as a function of the p_short_ - p_long_ parameter space. The arrows depict the effect of decreasing the p_short_ value at constant p_long_ and of decreasing the p_long_ value at constant p_short_, underscoring that compaction occurs only if the short-range looping probability is decreased, whereas a reduction of p_long_ results in de-compaction. The qualitative explanation for this behaviour is explained in the Discussion. Evidently, the DL-model model analysis does not intend to reflect the precise behaviour of interphase chromosomes. It shows how different looping regimes may dramatically affect large scale chromosome folding. As can be seen in Fig. 5 the behaviour is qualitatively the same over a large range of the p_long_ and p_short_ parameter landscape. Polymer modelling shows that the chromatin compaction we observe after knocking down CTCF and cohesin may be attributed to a specific reduction of short-range loops.

### Domain substructure of chromosomes

The adapted DL model does not incorporate what seems to be a key aspect of chromatin architecture, namely the presence of topological sub-chromosomal domains. Several independent lines of evidence, including replication timing, HiC analysis, clustering of epigenetic marks and electron microscopy, indicate that chromosomes of higher eukaryotes consist of arrays of topological domains in the size 0.5 to 5 Mb range [7]–[10]. In terms of the adapted DL model parameters this means that short-range looping interactions are locally clustered at many positions along the polymer, rather than evenly distributed as assumed in the adapted DL model. We wondered whether a polymer consisting of an array of topological domains would recapitulate the chromatin compaction we observe in our experiments when reducing the looping probability. To force the adapted DL model polymer to mimic topological domains we assumed that the polymer consists of regions that alternatingly contain a 50 monomer domain with high short-range looping probability (p_short_) and one that is devoid of short-range loops. For simplicity, in this domain-adapted DL model the two types of regions were made equal in length (50 monomers) resulting in 10 highly and 11 lowly looped domains in the model polymer of again in total 1050 monomers. Long-range loops were allowed between the compact domains only with a probability p_long_. Figure 3C shows the characteristic domain-like structure of the domain-adapted DL polymer under conditions of high short-range looping probability (p_short_ = 0.16) in combination with a low long-range probability (p_long_ = 0.02). As expected, compact domains (red in Figure 3C) are formed and connected by non-structured linker stretches (blue). When lowering the looping probability in the domains to p_short_ = 0.02 and increasing the looping between the domains (p_long_ = 0.16), the less compact individual domains (red) cluster due to long-range looping (Figure 3D). The heat map of the variation of polymer compaction in the p_short_ - p_long_ parameter space is shown in Figure 5B. Comparison of Figures 5A and B shows that the behaviour of polymers with and without domain-structure is very similar after decreasing short-range or the long-range looping probabilities. The former induces compaction and the latter results in expansion of the polymer. Evidently, introducing discrete topological domains in the adapted DL polymer model does not affect its capacity to show compaction after decreasing the short-range looping probability p_short_.

## Discussion

The 46 human chromosomes, in total consisting of 5 cm nucleosomal fibre, are packed in an interphase nucleus with a diameter in the order of 10 µm. Intra-chromosomal interactions forming chromatin loops play an important role in compacting interphase chromosomes. At the same time loops are a key component in gene regulation, for instance because they allow physical contacts between distant regulatory elements, such as promoters and enhancers [11]. Our understanding of basic principles of chromosome folding and how these affect gene regulation and other genomic functions is still limited. Polymer modelling based on experimental data sets begins to unveil general aspects of large scale (supra Mb) chromosome folding. The Dynamic Loop (DL) model shows that intra-chromosomal loops can explain the relatively compact nature of chromosome territories in interphase nuclei and why they intermingle only to a limited extent. Also, variations in looping frequency along the chromosomal fibre result in sub-chromosomal domains with different compaction [25]–[28].

All polymer models that are based on chromosome looping [16], [49] predict that a decrease in the number of loops results in chromosome expansion. Here we test this prediction in primary human fibroblasts by reducing the concentration of two abundant proteins that are involved in loop formation, i.e. CTCF and cohesin. Knocking down individually or simultaneously the CTCF gene and a gene coding for Rad21, an essential component of cohesin, reduces their concentration to about 20% of the initial levels (Figure 1). Others have shown that such decrease of CTCF and cohesin concentrations indeed results in a decrease in the number of chromosomal loops [36]–[38].

Remarkably, instead of the predicted expansion we observe a significant condensation of chromosome, in full contrast to the model predictions (Figure 2). These results confirm the importance of looping in controlling how chromosomes are folded. At the same time they put severe constraints on looping-based polymer models that should recapitulate the configuration of interphase chromosomes. Because the part of the binding sites on the genome of CTCF and cohesin co-localise [34], [35], it can be expected that knocking down of each of these proteins individually will affect similar effects, whereas their simultaneous knock down has a larger effect. This is exactly what is observed (Figure 2C–F).

To explain our observations we started from the DL model, which assumes that loops with a broad length distribution are randomly positioned along the chromosomal fibre. Systematic model simulations showed that selectively decreasing the frequency of short loops, without affecting longer loops, recapitulates the chromatin compaction observed in our experiments after decreasing the looping frequency by reducing the cellular concentration of CTCF and cohesin (Figures 4A, B and 5A). To do so we introduced separate parameters for the probability of short loops and of long loops (shorter than 5% of the polymer length and 5% or longer, respectively). Systematic simulations exploring variations of polymer compaction in the short-range vs. long-range looping probabilities show that only in a specific part of the parameter space a decrease of short-range looping frequencies results in significant condensation of the polymer. In contrast, decrease of long-range looping frequencies always results in decondensation.

**Figure 6:**
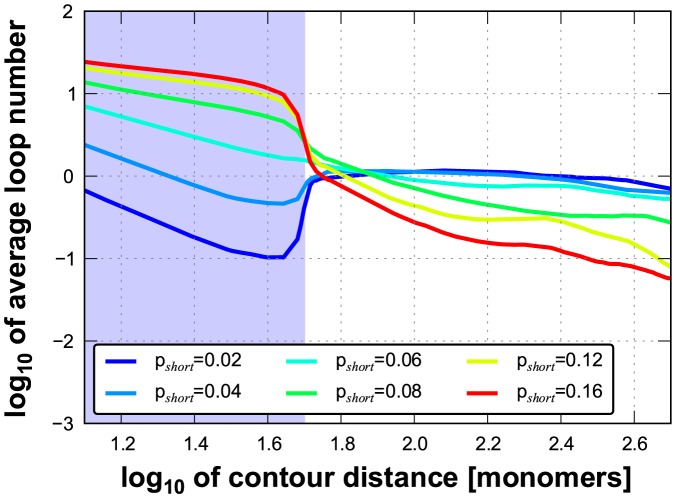
Relationship between looping probability and number of established loops. When keeping the long-range looping probability (p_long_) constant while varying short-range looping probability (p_short_), the number of long-range loops (n_long_) increases when reducing p_short_. This is because lowering p_short_ reduces the loop repulsion on the short scale and hence increases intermingling on the long scale, which in turn increases the frequency of long-range loop formation. Here, p_long_ = 0.06 and p_short_ is varied from 0.02 to 0.16. The blue area marks the short-range looping regime (<50 monomers) while the rest describes the long-range looping.

The observed chromatin compaction after decreasing the number of loops is counter-intuitive. The rationale is that reducing the number of short loops leads to expansion of the polymer on the short scale, resulting in an increase in volume occupied by the polymer. However, at the same time the decrease of the number of short loops leads to reduced entropic intra-polymer repulsion [27]. Consequently, parts of the polymer that are in close spatial proximity, e.g. due to the long-range interactions, intermingle stronger, which in turn makes the formation of a long-range loops more probable. This means that for constant p_long_ the number of long-range loops (n_long_) indirectly increases when reducing the number of short-range loops (Figure 6). As the entropy-driven volume reduction, due to this intra-polymer intermingling, exceeds the increase of volume due to the short-range polymer swelling, the volume occupied by the complete polymer decreases, i.e. it condenses. It may be argued that after de-compaction of the polymer at low length scales the probability of long range interaction increases because more such interaction sites become available. As can be seen in Fig. 5 such increase of p_long_ would further enhance the compaction process.

The above considerations predict that CTCF and cohesin are mainly involved in short-range looping. In support of this, Handoko et al. [48] showed that CTCF is predominantly engaged in the Mb-range organisation of chromosomes. Cohesin and CTCF are known to cooperate in the spatial organisation of chromosomes [34], [35]. In line with this, decreasing the activity of CTCF and cohesin preferentially affects looping at short-range, below Mb [36]–[38].

There is ample experimental evidence that, in contrast to what the adapted DL model assumes, the distribution of short-range loops is not random. Rather, short-range looping sites seem to cluster at multiple sites along the chromosome, resulting in topological domains in the 0.5–5 Mb size range [7]–[10]. Examples are distinct sub-chromosomal domains that differ in replication timing and in epigenetic state [2], [9], [12]–[15]. To mimic these topological domains in the adapted DL model, the short-range loops were clustered at multiple domains along the polymer, intervened by areas devoid of looping. Model simulations show that this redistribution of loops has little effect on the condensation-decondensation behaviour of the polymer (Figure 5B). Evidently, the domain-adapted DL polymer model reproduces the condensation of the polymer after reducing the number of short loops similar to the adapted DL model without domains (Figure 5A). Interestingly, after knockdown of CTCF and cohesin the Mb-size topological domains do not dissolve, indicating that at least part if the intra-domain loops remain intact. Zuin et al [36] showed that the number of interactions between chromatin from different topological domains, i.e. long-range loops, increases. As can be seen in Figure 5, the adapted DL model predicts that in addition to the entropic effects the increase of the frequency of large loops further contributes to polymer compaction.

In recent years, several polymer models have been proposed for the folding of interphase chromatin. The ‘Strings and Binders Switch’ (SBS) model of Barbieri et al. [49]), which assumes a diffusible component responsible for loop formation by linking two monomers of the polymer, is a special case of the DL model [16], [25]. In the DL model the properties of such ‘binders’ are incorporated in the looping probability parameter. As expected, the SBS model correctly mimics the formation of chromosome territories in the same way as the DL model. Another class of polymer models describes chromatin as a fractal polymer [22], [23]. This type of model was proposed to explain the chromatin folding based on HiC contact maps in the 0.5–10 Mb range [22], [50]. Since these models do not involve polymer looping-related parameters in their present form, they do not allow making predictions about how changes in the looping regime relate to changes in compaction. Systematic quantitative FISH measurements on chemically fixed cells, as used in this and other papers [16], [49], [51], do not support fractal models.

We can only speculate about the biological relevance of chromatin compaction after decreasing the concentration of the looping proteins CTCF and cohesin. Conceivably, this may be a step in the transition to metaphase chromosomes at the onset of cell division. A recent genome-wide interactome study shows that there are marked changes in intra-chromosomal contacts in metaphase chromosomes compared to the situation in interphase [52]. Whatever the mechanism of metaphase chromosome formation is, it most likely involves the disappearance of long-range loops. The inset in Figure 3A suggestively shows that complete abolishment of long-range loops results in metaphase-like structures in a state before axial condensation.

Taken together, the adapted DL model is successful in predicting various key properties of interphase chromosomes: (i) distinct and poorly intermingling chromosome territories, (ii) local differences in chromatin compaction along the chromosomal fibre, and (iii) chromosome compaction after reducing the looping frequency. The model shows that chromosomal looping constitutes the basis for this behaviour. Relatively subtle changes in the balance between short-range and long-range looping probabilities lead to changes in local and overall chromosome folding and compaction, probably including the interphase-metaphase transitions.

## Materials and Methods

### Cell culture

Human primary female fibroblasts (04–147) were cultured in DMEM containing 10% FCS, 20 mM glutamine, 60 µg/mL penicillin and 100 µg/mL streptomycin (Gibco, Life Technologies Corporation, Carlsbad, CA, USA). Cells were used up to passage 25 to avoid effects related to senescence.

### RNA interference

siRNA transfections were performed using lipofectamine RNAiMAX (Invitrogen, Life Technologies Corporation, Carlsbad, CA, USA) according to the reverse transfection protocol of the manufacturer. siRNAs used are listed in Table S2. The transfection efficiency of siRNA was estimated with the BLOCK-iT Alexa FluorRed Fluorescent Control (Invitrogen, Life Technologies Corporation, Carlsbad, CA, USA) in three independent experiments. In each experiment 100 nuclei were scored based on DAPI signal, 99% of which showed red fluorescence and therefore had been transfected successfully. Knockdowns were verified by Western blot. Cells were resuspended in lysis buffer (10 mM HEPES, 10 mM KCl, 1,5 mM MgCl_2_, 0,34M sucrose, 10% glycerol, 1 mM DTT, complete protease inhibitor cocktail (Roche, F. Hoffmann-La Roche Ltd, Basle, Switzerland)) containing 0.1% Triton X-100, incubated 10 min on ice and centrifuged 5 min at 1300×g. Pellets were resuspended and washed once. We used anti-CTCF polyclonal antibody (07-729) from Millipore (Millipore, Temecula, CA, USA) and anti-Rad21 polyclonal antibody (abb992) from Abcam (Abcam plc, Cambridge, UK), both in 1∶1000 dilutions to detect CTCF and Rad21 protein levels. As a control antibody against β-Actin was used - anti-β-Actin (A1978) from Sigma-Aldrich (Sigma-Aldrich, Saint Louis, MO, USA) in 1∶5000 dilution. Secondary antibodies: AP-conjugated Anti-Rabbit IgG (111-055-003) and AP-conjugated Anti-Mouse IgG (115-055-003) both from Jackson (Jackson ImmunoResearch Laboratories Inc, West Grove, PA, USA) were used in dilution 1∶5000. Signals were quantified using ImageJ software (http://rsb.info.nih.gov/ij/).

### Measuring apoptotic cells

The fraction of apoptotic cells on slides was estimated by FITC Annexin V (BD Pharmingen, Becton, Dickinson and Company, Franklin Lakes, NJ, USA) staining. 100 cells of control, CTCF knockdown, Rad21 knockdown and CTCF-Rad21 knockdown populations were selected by DAPI signal, the fraction of Annexin V positive cells was measured in this population. The same procedure was repeated for three independent siRNA knockdown experiments. Results obtained by AnnexinV staining were confirmed by the Tunel apoptosis assay (Promega, Madison, WI, USA).

### Labelling of BAC probes and fluorescence in situ hybridization

BACs were selected from the BAC clones available in the RP11-collection at the Sanger Institute. Genomic distances were defined as the distance between centres of the BACs. BAC DNA was isolated using the Qiagen REAL prep 96 kit (Qiagen, Qiagen Benelux BV, Venlo, Netherlands). Nick-translation was used to label the probes, either with digoxigenin or biotin (Roche, F. Hoffmann-La Roche Ltd, Basle, Switzerland). FISH was carried out as described in [44]. FITC-conjugated antibodies (Roche, F. Hoffmann-La Roche Ltd, Basle, Switzerland) and Cy3-conjugated streptavidin (Jackson ImmunoResearch Laboratories, Inc., West Grove, PA) were used to visualize the hybridization signals. DAPI (4′,6′-diamidino-2-phenylindole) (Sigma-Aldrich, Saint Louis, MO, USA) was used to outline the cell nucleus.

To exclude S-phase cells from the analysis the culture was incubated with bromodeoxyuridine (BrdU) to label replicating cells 30 min before fixation, followed by immunolabelling in combination with FISH labelling as described in [44].

### Imaging

Twelve-bit 3D images were recorded using Nikon A1R confocal laser-scanning microscope (Nikon, Tokyo, Japan) equipped with a 100×/1.49 NA Apo TIRF DIC objective (Nikon), using a diode laser at 405 nm, an Ar laser at 488 nm and a diode-pumped solid-state laser at 561 nm to excite DAPI, FITC and Cy3, respectively. Fluorescence was detected with the following bandpass filters: 425–475 nm (DAPI), 500–550 nm (FITC) and 570–620 nm (Cy3). Images were scanned with a voxel size of 60×60×100 nm.

### Image processing and data analysis

All confocal images were subject to deconvolution using Huygens Professional 3.7 software (Scientific Volume Imaging, Hilversum, The Netherlands) using the measured point spread function (PSF) for each channel and the classical maximum likelihood estimation algorithm. The PSF was obtained by imaging Tetraspeck Fluorescent Microsphere Standards with a diameter of 200 nm (Invitrogen). The signal to noise ratios and background intensities were estimated for each channel and averaged from several images. These values were used as a standard for batch processing deconvolution of all the image stacks.

Automated image analysis was carried out on deconvolved datasets with the ARGOS software (http://homepages.cwi.nl/~wimc/argos) to identify nuclear sites labelled by BACs and to compute their 3D position in the nucleus as described in [44]. Chromatic aberration was measured via Tetraspeck Fluorescent Microsphere Standards with a diameter of 200 nm (Invitrogen) and corrected for in the analysis. After background subtraction, images were treated with a bandpass filter to remove noise. Subsequently, images were segmented and ensembles of interconnected voxels were regarded as the site labelled by a BAC. The centre of mass was calculated for each labelled site at a sub-voxel resolution and 3D distances between BACS were measured. To estimate the systematic measuring error we hybridized cells with a mixture of the same BAC marked with two different fluorophores and measured the distances between the two signals. Accuracy of measurements was better than 50 nm in three dimensions.

The radial nuclear position *p_n_* (*p_n_* = *r_o_/r_n_*) of BAC probe indicates positioning of the centre of gravity of a FISH labelled site on the line drawn from the centre of gravity of the nucleus to nuclear envelope. *p_n_* was calculated as previously described [44]: the distance from the centre of gravity of a BAC probe and the centre of the nucleus (*r_o_*) was divided by the length of a line from the nuclear centre to the nuclear envelope through the centre of gravity of the BAC probe (*r_n_*). *p_n_* value close to 1 indicates positioning of the BAC probe at nuclear periphery while *p_n_* value close to 0 indicates its positioning close to the centre of gravity of the nucleus.

### Dynamic Loop polymer model

Chromatin fibres are in general modelled as un-branched polymer chains. The modelled polymers are coarse-grained versions of the real chromosomes, where each monomer represents a stretch of DNA. If the length of this stretch is much larger than the persistence length of the chromatin fibre, we can expect the polymer to be totally flexible and are allowed to neglect the influence of the local bending rigidity. With a persistence length below 250 nm [53] and a fibre packing, where a length of 10 nm = 1 kb, the monomers should represent DNA stretches with a length of at least 50 kb.

In this study, we performed Monte-Carlo simulations [54] with an implementation of the Dynamic Loop model to generate chromosomal conformations. For the polymer chains that represent the interphase chromosomes we used the well-tested bond fluctuation method [55], [56] to investigate their structure, as well as their dynamics. In the simulations a monomer of the polymer chain is randomly selected and, if possible, randomly moved to one of its nearest neighbours on the lattice. Excluded volume interactions are taken into account by preventing a lattice site to be occupied by more than one monomer. When simulating N monomers we define one Monte-Carlo step (MCS) to correspond to N moves, i.e. on average each monomer is translated once during a MCS. Due to the fact that the polymer conformations only exhibit slight changes from one MCS to the next, the time span when two conformations can be considered to be independent must be determined. Therefore, we calculate the autocorrelation function of the polymer's squared radius of gyration Rg2(t) which is a measure for structural correlation. We obtain the estimated autocorrelation time τ_ac_ by applying an exponential fit to the autocorrelation function. Finally, we set 5τ_ac_ MCS as the time span above which two conformations are expected to be independent.

### Chromatin looping

For the interactions between the chromatin fibres we used the Dynamic Loop (DL) model [25]. In this model two monomers that are non-adjacent along the fibre are only allowed to interact if they are in spatial proximity, i.e. if the distance between them is below a certain cutoff. When two monomers i and j approach each other due to diffusional motion and the cutoff condition is fulfilled, a bond can be established between them with a certain probability p_bond,ij_, the binding or looping probability. In case the bond is formed, a lifetime t_bond_ chosen from a Poisson distribution with mean T_bond_ is assigned to it, determining when the bond dissociates. Hence, the bonds can frequently change during the course of the simulation, which mimics the effect of the highly dynamic DNA-DNA interactions. In contrast to this, the bonds along the backbone of the polymer are fixed and cannot break open.

### Polymer model simulations

For the simulations with the domain-adapted DL and with the adapted DL model, the chromosome-representing polymers have a length of 1050 monomers. We have to simulate thousands of independent chromosome conformations for each parameter set to get statistically reliable values for the different chromosome properties. Hence, the polymer length is limited to ∼1000 monomers in order to finish the simulations in a manageable timeframe. We set the length to exactly 1050 monomers because in the domain-adapted DL model, the highly active as well as the lowly active domains have a size of 50 monomers and the polymer consists of 21 domains (11 lowly active interspersed by 10 highly active regions). We also use 1050 monomers for the adapted DL model to ease comparison with the results from the domain-adapted DL model. The size of the Monte-Carlo lattice is set to a large value (L = 500) to avoid interactions caused by periodic boundary conditions. The mean of the Poisson distributions that is used to determine the bond lifetime is set to 8000 MCS. In the adapted DL model, p_bond,ij_ can take 2 different values, namely p_short_ if |i-j|< = 50 and p_long_ otherwise. In the domain-adapted -DL model, the bond formation probabilities p_bond,ij_ between the monomers are set to p_short_ if monomers i and j are inside the same domain, or to p_long_ if i and j belong to different domains.

We start each simulation by creating a random self-avoiding walk (polymer where bonds are only established between adjacent monomers. We continue with a first equilibration run of 10^8^ MCS where only homogeneous bond formation is allowed. As p_bond_ is set to be constant along the fibre, we obtain a homogenous starting conformation, meaning a polymer with a uniform structure. In a second equilibration run of 10^8^ MCS we allow heterogeneous looping as defined by the domain-adapted DL and the adapted DL model. When all equilibration runs are finished, the main simulation starts and the chromosomal conformation is saved every 10^7^ MCS. This is done until at least 1000 independent conformation are generated.

## Supporting Information

Figure S1 **Cellular and nuclear morphology of cells depleted in CTCF and/or cohesin.** Phase contrast images (A) and images of DAPI stained cells with visible FISH probes (B) showing 04-147 primary fibroblasts before and after siRNA mediated depletion of CTCF, Rad21 and simultaneous depletion of CTCF and Rad21. For (B) deconvolved 3D confocal images were flattened using ImageJ. DAPI signal is a standard deviation projection over all stacks, FISH signals are maximum intensity projections.
(TIF)

Figure S2 **Nuclear size of cells depleted in CTCF and/or cohesin.** 3D imaged nuclei were automatically detected and their volume measured in ARGOS software (http://homepages.cwi.nl/~wimc/argos). Box plot shows volumes of at least 250 nuclei per condition. Outliers are indicated in red. There is no statistical difference between datasets obtained from control cells and cells depleted in CTCF, Rad21 or CTCF and Rad21 (t-test).
(EPS)

Figure S3 **Fraction of apoptotic cells in experiments.** Fraction of apoptotic cells on slides was estimated by FITC Annexin V staining. 100 cells of control, CTCF knockdown, Rad21 knockdown and CTCF-Rad21 knockdown populations were selected by DAPI signal, the fraction of Annexin V positive cells was measured in this population. The same procedure was repeated for three independent siRNA knockdown experiments. Error bars represent standard deviation.
(EPS)

Figure S4 **FISH measurements of cells transfected with non-target siRNA.** To test whether the siRNA transfection process itself affects chromatin compaction we carried out FISH measurements on cells transfected with 150 nM non-target siRNA (Negative Control Medium GC Duplex, Invitrogen). Transfections were carried out as in the case of CTCF Rad21 double depletion. Error bars represent standard error of mean.
(EPS)

Figure S5 **Example of polymer conformations of the adapted DL model at different looping regimes.** (A) Conformation of a adapted DL polymer with high short-range (pshort = 0.12) and low long-range (plong = 0.04) looping probabilities and (B) the same polymer with low short-range (pshort = 0.04) and high long-range (plong = 0.12) looping probabilities. The colour code labels the monomers of the polymer according to the visible spectrum.
(EPS)

Figure S6 **Genomic alignment of transcriptome maps, replication domains, topological domains data, and BAC probes.** Alignment of the transcriptome map (04-147 primary fibroblasts [42]), replication timing domains (IMR90 foetal lung fibroblasts [43]), coordinates of HiC topological domains (IMR90 [7]) and the positions of BAC probes used in this study (A) for regions analysed on Chromosome 1 and (B) for the region on Chromosome 11. On the human transcriptome map vertical lines represent protein coding genes, the length depicting the average transcription level in a number of human tissues and cell lines. Replication-timing domains are illustrated by peaks and valleys corresponding to early and late-replicating regions, respectively. Coordinates of topological domains identified using HiC are shown as blocks (HiC data processing described in reference [7]). For better visibility of domain borders alternating blocks have different height; regions devoid of blocks are linker areas between domains. Triangles below the plot indicate positions of BAC probes. Reference probes for each dataset are indicated in red. The same probes are indicated above the graph of the transcriptome map by bars (as on figure 2). Gene-rich and gene-poor genomic regions analyzed are indicated by green and red bar, respectively.
(EPS)

Table S1 **BACs used and their positions on human genome assembly 18 (Hg18).**
(DOCX)

Table S2 **siRNAs used.**
(DOCX)
