# Correction: Depletion of the Chromatin Looping Proteins CTCF and Cohesin Causes Chromatin Compaction: Insight into Chromatin Folding by Polymer Modelling

**DOI:** 10.1371/journal.pcbi.1004716

**Published:** 2016-01-15

**Authors:** The PLOS Computational Biology Staff

Ingrid M. van der Wateren should be included as a co-author of this paper.

The author’s affiliation is as follows:

Swammerdam Institute for Life Sciences, University of Amsterdam, Amsterdam, The Netherlands

The author list should read as follows:

Mariliis Tark-Dame, Hansjoerg Jerabek, Erik M. M. Manders, Ingrid M. van der Wateren, Dieter W. Heermann, Roel van Driel

The author contributions section should read as follows:

Conceived and designed the experiments: RvD DWH MTD HJ.

Performed the experiments: MTD HJ IMvdW.

Analyzed the data: MTD HJ IMvdW.

Contributed reagents/materials/analysis tools: EMMM.

Wrote the paper: MTD RvD HJ DWH.

The citation for this paper should be as follows:

Tark-Dame M, Jerabek H, Manders EMM, van der Wateren IM, Heermann DW, van Driel R (2014) Depletion of the Chromatin Looping Proteins CTCF and Cohesin Causes Chromatin Compaction: Insight into Chromatin Folding by Polymer Modelling. PLoS Comput Biol 10(10): e1003877. doi:10.1371/journal.pcbi.1003877

The authors confirm that the addition of this co-author does not affect the competing interest statement or funding statement.
