# Supplementary figures and images for: Depletion of the Chromatin Looping Proteins CTCF and Cohesin Causes Chromatin Compaction: Insight into Chromatin Folding by Polymer Modelling
Source: PLoS Comput Biol. 2014 Oct 9;10(10):e1003877. doi: 10.1371/journal.pcbi.1003877 (PMC4191888; doi:10.1371/journal.pcbi.1003877)

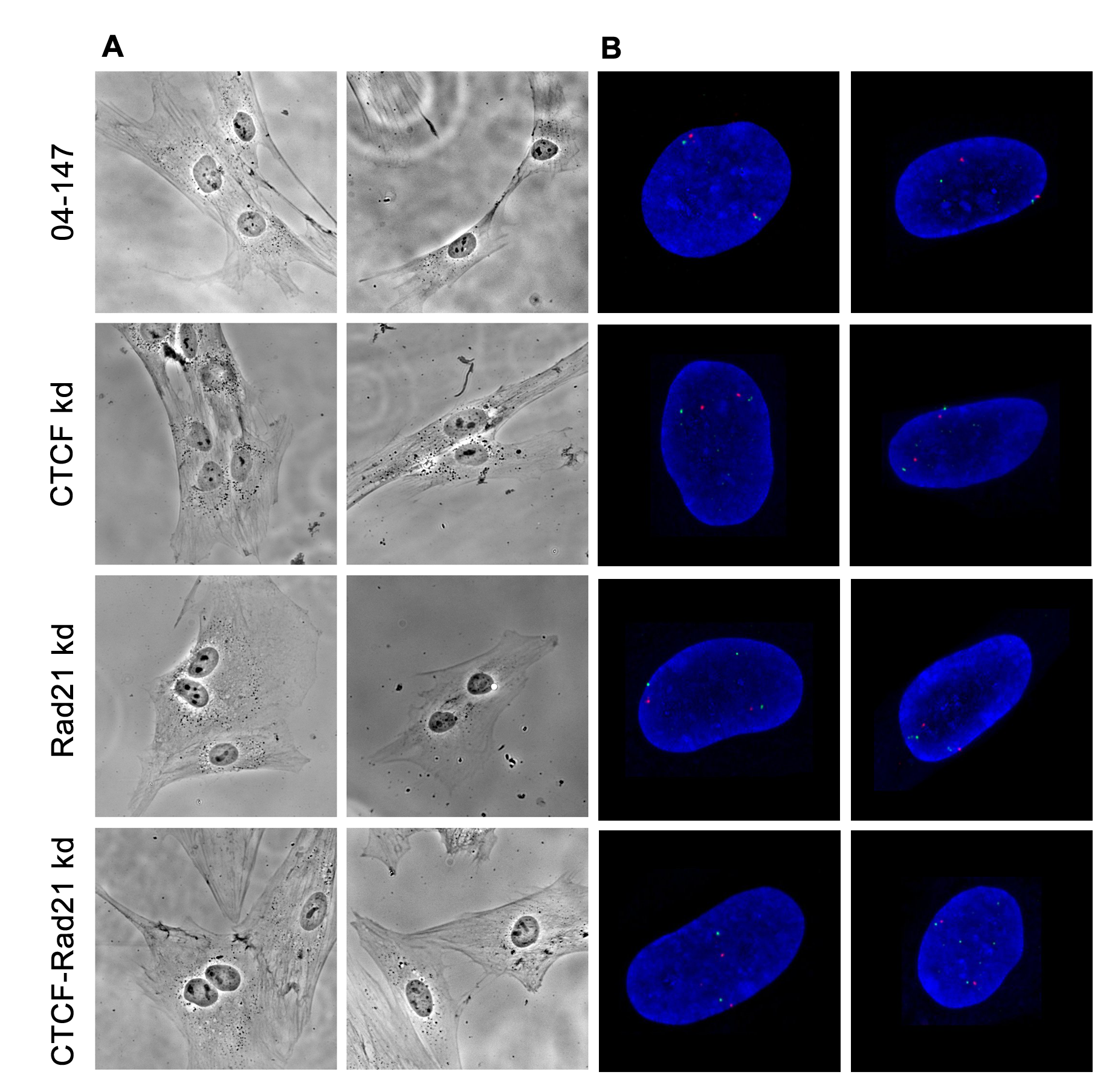

Supplement: Figure S1 — Cellular and nuclear morphology of cells depleted in CTCF and/or cohesin. Phase contrast images (A) and images of DAPI stained cells with visible FISH probes (B) showing 04-147 primary fibroblasts before and after siRNA mediated depletion of CTCF, Rad21 and simultaneous depletion of CTCF and Rad21. For (B) deconvolved 3D confocal images were flattened using ImageJ. DAPI signal is a standard deviation projection over all stacks, FISH signals are maximum intensity projections. (TIF) [file pcbi.1003877.s001.tif]
