# Supplementary material for: Depletion of the Chromatin Looping Proteins CTCF and Cohesin Causes Chromatin Compaction: Insight into Chromatin Folding by Polymer Modelling
Source: PLoS Comput Biol. 2014 Oct 9;10(10):e1003877. doi: 10.1371/journal.pcbi.1003877 (PMC4191888; doi:10.1371/journal.pcbi.1003877)
Supplement: Table S1 — BACs used and their positions on human genome assembly 18 (Hg18). (DOCX) [file pcbi.1003877.s007.docx]

**Table S1. BACs used and their positions on human genome assembly 18 (Hg18).**

| **BAC** | **Chr** | **Start** | **End** | **Distance (Mbp)*** | **number of distances analysed** | | | |
| --- | --- | --- | --- | --- | --- | --- | --- | --- |
| **Chromosome 1** | | | | | **Control 04-147** | **CTCF kd** | **Rad21 kd** | **CTCF-Rad21 kd** |
| **Gene-poor region** | | | | | | | | |
| **RP11-252L24** | **chr1:** | **174797244** | **174978591** | **Reference** | | | | |
| RP11-655F10 | chr1: | 174977032 | 175146843 | 0,17 | 54 | 23 |  | 42 |
| RP11-195J4 | chr1: | 175300547 | 175451086 | 0,49 | 64 | 72 |  |  |
| RP11-524J18 | chr1: | 175617024 | 175789954 | 0,82 | 149 | 30 | 62 | 34 |
| RP11-142H13 | chr1: | 176124727 | 176312221 | 1,33 | 42 | 46 | 152 | 62 |
| RP11-162H11 | chr1: | 176574921 | 176730902 | 1,76 | 39 | 72 | 112 | 48 |
| RP11-177A2 | chr1: | 177196859 | 177408098 | 2,41 | 47 | 21 | 41 | 68 |
| RP11-231P5 | chr1: | 177764373 | 177908342 | 2,95 | 49 | 84 | 52 | 93 |
| **Gene-rich region** | | | | | | | | |
| **RP11-77D16** | **chr1:** | **155356363** | **155533386** | **Reference** | | | | |
| RP11-341K5 | chr1: | 155185068 | 155397482 | 0,15 | 36 | 140 | 90 | 59 |
| RP11-173A2 | chr1: | 155029936 | 155216620 | 0,32 | 50 | 48 | 44 | 57 |
| RP11-139I14 | chr1: | 154512452 | 154689574 | 0,84 | 29 | 78 | 45 | 193 |
| RP11-552H7 | chr1: | 154136195 | 154277806 | 1,24 | 60 | 62 | 67 | 65 |
| RP11-653F24 | chr1: | 153502717 | 153653162 | 1,87 | 60 |  | 53 |  |
| RP11-624P9 | chr1: | 153079766 | 153230241 | 2,29 | 56 | 72 | 25 | 35 |
| RP11-196D4 | chr1: | 153023104 | 153200297 | 2,33 | 50 | 74 | 26 | 20 |
| RP11-749D14 | chr1: | 152524737 | 152689783 | 2,84 | 55 | 70 | 53 | 17 |
| **Long distance region** | | | | | | | | |
| **RP11-68O15** | **chr1:** | **151672468** | **151771086** | **Reference** | | | | |
| RP11-756M22 | chr1: | 153541678 | 153692169 | 1,9 | 81 | 62 | 40 |  |
| RP11-217A13 | chr1: | 155661462 | 155823045 | 4,02 | 61 | 55 | 55 | 72 |
| RP11-10P13 | chr1: | 157766153 | 157924825 | 6,12 | 115 | 53 |  |  |
| RP11-464H15 | chr1: | 159778965 | 159996454 | 8,17 | 48 | 62 | 37 | 25 |
| RP11-124M2 | chr1: | 161777331 | 161938456 | 10,14 | 33 | 35 | 55 | 60 |
| RP11-92H17 | chr1: | 165758164 | 165946922 | 14,13 | 24 | 37 | 57 |  |
| RP11-212H21 | chr1: | 167626665 | 167785984 | 15,98 | 39 | 72 | 47 | 72 |
| RP11-242I9 | chr1: | 169682353 | 169832450 | 18,04 | 54 | 50 | 35 | 28 |
| RP11-136P6 | chr1: | 171773860 | 171938712 | 20,13 | 75 | 35 |  | 60 |
| RP11-252L24 | chr1: | 174797244 | 174978591 | 23,17 | 102 | 48 | 51 | 20 |
| RP11-142H13 | chr1: | 176124727 | 176312221 | 24,5 | 53 | 28 |  | 42 |
| RP11-620B8 | chr1: | 177945800 | 178103190 | 26,3 | 41 | 94 | 68 | 66 |
|  | | | | | | | | |
| **Chromosome 11** | | | | | | | | |
| **RP11-158E6** | **chr11:** | **58087310** | **58252490** | **Reference** | | | | |
| RP11-467L20 | chr11: | 61203193 | 61391707 | 3,13 | 77 |  | 40 | 91 |
| RP11-472L10 | chr11: | 63363052 | 63485403 | 5,25 | 67 | 70 | 86 | 67 |
| RP11-300I6 | chr11: | 69162461 | 69323966 | 11,07 | 40 | 48 | 77 | 63 |
| RP11-449K13 | chr11: | 81088431 | 81193431 | 22,97 | 52 | 56 | 71 |  |
| RP11-126P16 | chr11: | 89926738 | 90087711 | 31,84 |  | 47 | 54 |  |
| RP11-2I10 | chr11: | 98817916 | 98971673 | 40,72 | 72 | 26 | 39 | 27 |
| RP11-39H20 | chr11: | 108478663 | 108665432 | 50,4 | 28 | 30 | 32 | 32 |
| RP11-469N6 | chr11: | 133984001 | 134156378 | 75,9 | 35 | 38 | 54 | 48 |

reference BACs are in bold. * Distance from respective reference BAC, measured between centres of respective BAC sequences.
