# Supplementary material for: Depletion of the Chromatin Looping Proteins CTCF and Cohesin Causes Chromatin Compaction: Insight into Chromatin Folding by Polymer Modelling
Source: PLoS Comput Biol. 2014 Oct 9;10(10):e1003877. doi: 10.1371/journal.pcbi.1003877 (PMC4191888; doi:10.1371/journal.pcbi.1003877)
Supplement: Table S2 — siRNAs used. (DOCX) [file pcbi.1003877.s008.docx]

**Table S2. siRNAs used.**

| **siRNA** | **catalogue  number** | **Sequence / Description** | **final concentration** | **duration of knockdown** |
| --- | --- | --- | --- | --- |
| CTCF | CTCFHSS116455 (Invitrogen) | UCA CCC UCC UGA GGA AUC ACC UUA A  UUA AGG UGA UUC CUC AGG AGG GUG A | 100nM | 72h |
| Rad21 | RAD21HSS109005 (Invitrogen) | AGA GUC UGA ACA GAG CAC CAG CAA U  AUU GCU GGU GCU CUG UUC AGA CUC U | 50nM | 48h |
| Positive control | 14750100  (Invitrogen) | BLOCK-iT Control Alexa Fluor® Red Fluorescent | 50nM | 24h |
| Negative control | 12935300  (Invitrogen) | Negative Control Medium GC Duplex, | 150nM | 72h |
